# Global scoping review of HIV prevention research with transgender people: Transcending from trans‐subsumed to trans‐centred research

**DOI:** 10.1002/jia2.25786

**Published:** 2021-09-02

**Authors:** Ana María del Río‐González, María Lameiras‐Fernández, Djordje Modrakovic, Rodrigo Aguayo‐Romero, Courtney Glickman, Lisa Bowleg, Maria Cecilia Zea

**Affiliations:** ^1^ Department of Psychological and Brain Sciences The George Washington University Washington District of Columbia USA; ^2^ Facultad de Ciencias da Educación Universida de Vigo Ourense Spain; ^3^ Brigham and Women's Hospital/Harvard Medical School/The Fenway Institute Boston Massachusetts USA

**Keywords:** HIV prevention research, scoping review, transgender people

## Abstract

**Introduction:**

Globally, transgender populations are disproportionally impacted by HIV and effective HIV prevention interventions targeting these populations are critically needed. Such interventions require research focused on the specific needs and experiences of transgender people. This methodological review aims to determine the extent to which HIV prevention research has included transgender participants by subsuming them into non‐transgender populations, or by centring them either in comparison with other groups or as the sole focus of research.

**Methods:**

We searched five electronic databases (e.g. SCOPUS) for empirical studies that focused on HIV prevention and included transgender participants, published through 31 December 2020. For each study, we extracted information on: (a) types of inclusion of transgender participants; (b) total sample size and number/percentage of transgender participants; (c) country(ies) where study was conducted; (d) HIV research topics; (e) methods (i.e. quantitative, qualitative or mixed‐methods research) and (f) gender identity of transgender participants.

**Results and discussion:**

Of 667 HIV prevention studies included in the review, 38.5% subsumed transgender participants into cisgender populations (most frequently combining transgender women with cisgender men who have sex with men), 20.4% compared transgender and cisgender participants and 41.1% focused exclusively on transgender populations. Our global scoping review also revealed that these three types of transgender inclusion in HIV prevention research vary greatly over time, place and thematic areas. Transgender women are the focus of the majority of reviewed studies, whereas transgender men and gender expansive people are rarely included as participants.

**Conclusions:**

Inclusion of transgender persons as participants in HIV prevention research has significantly increased, particularly in the last decade. Further research centred on transgender participants and their experiences are needed to develop effective HIV prevention interventions for transgender populations. We advocate for HIV prevention research to move from subsuming transgender people, to trans‐centred research that asks questions that focus on their specific needs and experiences. We provide recommendations to move from trans‐subsumed to trans‐centred HIV prevention research.

## 1 INTRODUCTION

‘Transgender’ is an umbrella term that describes people whose gender identity or expression is different from their sex assigned at birth. There are a myriad of terms to describe the vast diversity of gender identities and expressions (e.g. transgender women, transgender men, gender non‐binary). Transgender people share in common the ubiquity of the stigma and discrimination they face, and the associated social‐structural vulnerabilities, including lack of recognition of their right to access care and the absence of trans‐competent care services. These vulnerabilities disproportionately place transgender people at risk for multiple health‐related issues [1, 2]. In the case of HIV, although accurate estimates of prevalence are scarce due to the absence of gender identity indicators in HIV surveillance, a 2013 global meta‐analysis reported that transgender women were 49 times more likely to be HIV positive compared with all adults of reproductive age [3]. The limited existing data on transgender men and gender expansive people (i.e. those who do not subscribe to the masculine‐feminine gender binary, or who choose not to be defined by their gender) evidence an elevated HIV risk for these groups, particularly amongst those who have sex with men [4, 5, 6].

There have been several calls to study the specific factors that contribute to increased HIV vulnerability amongst transgender people. In the 2014 consolidated guidelines on HIV prevention, diagnosis, treatment and care for key populations, the World Health Organization (WHO) recognized that ‘the high vulnerability and specific health needs of transgender people necessitate a distinct and independent status in the global HIV response’ [7]. Multiple HIV strategies at the national and regional levels have echoed this recognition [8, 9, 10, 11]. As a result, inclusion of transgender people in HIV‐related research is increasingly receiving global attention, and several systematic reviews, meta‐analyses and other literature overviews have been published in this area [3, 4, 5, 12, 13, 14, 15, 16, 17, 18, 19, 20, 21, 22, 23, 24, 25, 26, 27, 28]. Less attention has been paid, however, to the ways in which transgender people are included as participants in HIV prevention research, the focus of this scoping review.

Incomplete knowledge about the health of a population impedes their inclusion in the public health agenda [29]. In her seminal essay on the politics of public health data, Nancy Krieger cautioned us about how the data we collect – or fail to collect – determines the health problems we recognize as relevant and deserving of intervention: ‘No data, no problem’ [30]. If HIV‐related research fails to include transgender participants, or makes them invisible as research participants, transgender populations are less likely to benefit from targeted HIV prevention programs and interventions. Previous reviews point out that HIV research frequently ignores transgender populations in two ways: firstly, by relying on assessments of biological sex and not gender identity, or secondly, by purposely excluding participants based on biological sex rather than gender identity and sexual behaviour [e.g. excluding transgender men who have sex with men (MSM) from studies of MSM].

### 1.1 Trans‐subsumed versus trans‐centred research

Including and identifying transgender participants in HIV prevention research is the first step to reduce the disproportionate burden of HIV amongst transgender populations. Nevertheless, even when transgender participants are included and identified during data collection, they can become invisible if researchers subsume them into other non‐transgender groups during data analysis (e.g. considering transgender women as MSM). A true understanding of the factors that heighten HIV risk amongst transgender people requires research that centres investigative endeavours on the specific experiences, needs and characteristics of transgender participants. Trans‐centred research can be conducted in two ways: by sampling transgender participants exclusively, or by including both transgender and cisgender participants, and asking trans‐specific questions and conducting trans‐specific analyses (e.g. group comparisons or moderation by gender identity).

This global scoping review assesses the extent to which HIV prevention research has included transgender participants in one of three ways: (1) as a group that is subsumed into non‐transgender populations (i.e. trans‐subsumed); (2) as a group that is compared with other groups (i.e. trans‐comparative); or (3) as the sole focus of research (i.e. trans‐exclusive). To provide a comprehensive map of the degree to which HIV prevention research is trans‐subsumed, trans‐comparative or trans‐exclusive, we examine how these forms of inclusion vary over time, place, thematic areas, methods and participants’ gender identities. We selected a scoping review of the literature because it would allow us to examine the extent, range and nature of available evidence in this broad topic area, rather than to answer a more precise question, which would require a systematic review [31, 32].

## 2 METHODS

Following guidelines for scoping reviews [31, 32], we conducted a systematic search to identify literature on HIV prevention research that has included transgender participants. We used a broad definition of HIV prevention research that includes not only the development and evaluation of preventive interventions [e.g. pre‐exposure prophylaxis (PrEP)], but also observational studies assessing factors associated with HIV prevalence (e.g. condomless sex) or with HIV prevention (e.g. attitudes towards condoms). We excluded studies that focused solely on the care of people living with HIV (e.g. treatment as prevention).

### 2.1 Search strategy and selection criteria

We searched Academic Search Complete, MEDLINE, PsycINFO, PubMed and SCOPUS. Search terms used were: (’HIV prevention’ OR ‘prevention of HIV’) AND (transgender* OR transsexual* OR ‘gender dysphoria’ OR ‘gender identity disorder’ OR genderqueer OR ‘gender queer’ OR transvesti* OR transwomen OR transmen OR ‘fa'afafine’ OR hijra OR kathoey OR waria OR muxe OR two‐spirit OR ‘two spirit’ OR ‘third gender’ OR ‘Gender diverse’ OR ‘gender non‐binary’ OR ‘gender non‐binary’ OR ‘gender expansive’). We did not impose any limitations regarding geographical location or language, but searches were conducted in English only. To locate additional relevant publications not identified during the database searches, we examined the reference lists of published reviews and conceptual papers in the field [3, 4, 5, 6, 11, 12, 13, 14, 15, 16, 17, 18, 19, 20, 21, 22, 23, 24, 25, 26, 27, 33, 34, 35, 36], and used Web of Science's citation tracking to find articles that had cited those reviews and conceptual papers. We conducted an initial search on March 2018, and then updated the search on April 2021 to include articles published until 31 December 2020.

Publications were deemed eligible for inclusion if they: (a) were published in a peer‐reviewed journal; (b) presented empirical findings focusing on any aspect of HIV prevention (e.g. sexual risk, HIV testing, condom use), except treatment as prevention; (c) included at least one transgender person as participant and (d) provided the number of transgender participants included.

### 2.2 Data analysis

The coding team consisted of the first five authors. As a first step, we identified and excluded duplicate articles. Next, the first author screened all articles at the title and abstract level for exclusion. For articles not excluded at this initial stage, the first author and one additional reviewer independently reviewed the full text for decisions regarding final inclusion. For included articles, reviewers used a standardized form to extract information on: (a) types of inclusion of transgender participants; (b) total sample size and number/percentage of transgender participants; (c) country(ies) where study was conducted; (d) HIV research topics; (e) methods (i.e. quantitative, qualitative or mixed‐methods research) and (f) gender identity of transgender participants. Disagreements regarding article inclusion and data extraction were resolved through discussion amongst the independent reviewers until we reached consensus.

We created three categories to classify studies regarding the ways in which they included transgender participants:

1. *Studies that subsumed transgender people into other groups (trans‐subsumed)*. Studies in this category included both transgender and cisgender (i.e. non‐transgender) participants but did not analyse data from transgender participants separately. For example, studies that included ‘transgender’ as a main effect variable in regression‐like analyses but did not perform any further analyses comparing transgender and cisgender participants (e.g. moderation of other effects by transgender identity) were included in this category.
2. *Studies that compared transgender people with other groups (trans‐comparative)*. Studies in this category included both transgender and cisgender participants as separate groups (e.g. transgender and cisgender sex workers), and either asked transgender‐specific questions or conducted at least one set of analysis comparing these groups.
3. *Studies that focused exclusively on transgender people (trans‐exclusive)*. Studies in this category sampled transgender participants only. Studies that also included information from key informants (e.g., healthcare providers) were included in this category, but key informants were not counted as participants or included in sample size.

To assess how these three types of inclusion of transgender participants varied over time, place, thematic areas, methods and gender identities of participants, we used Pearson's chi‐square tests in SPSS version 23. To analyse place variations in the ways in which transgender participants have been included in HIV prevention research, we grouped countries based on the UNAIDS regional classification [37]; due to the large number of USA‐based articles, we separated the USA from the ‘Western and Central Europe and North America’ region. We classified multiregional research (e.g. iPrEx study [38], conducted in Brazil, Ecuador, Peru, South Africa, Thailand and the USA) into a separate group.

Because of the diversity of HIV prevention topics covered in the studies, we created three broad categories of research areas: (1) psychological/behavioural, (2) biomedical/epidemiological and (3) social‐structural. Studies were categorized as psychological/behavioural if they included findings regarding individual‐level behaviours (e.g. condom use, HIV testing) or psychological factors (e.g. attitudes, self‐efficacy) related to HIV prevention. The second category included studies assessing biomedical [e.g. post‐exposure prophylaxis (PEP), PrEP] or epidemiological (e.g. prevalence, incidence) topics. The last category included research examining HIV‐related factors at the social (e.g. stigma, violence) and structural (e.g. policies, legislation) levels, including socio‐demographic characteristics (e.g. education, unemployment). Studies could be classified in more than one area; for example, a study assessing individual‐ and social‐level factors associated with PrEP use would be included in all three areas of research.

## 3 RESULTS AND DISCUSSION

**Figure 1:**
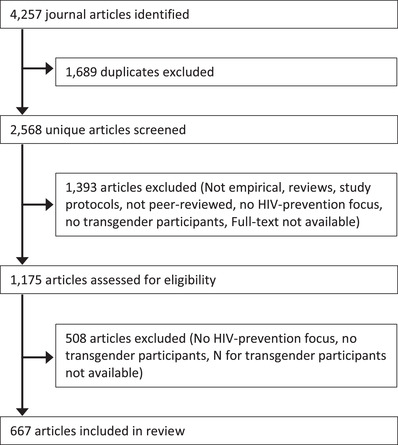
PRISMA flow diagram of article selection. Inclusion of transgender people as participants in HIV prevention research.

Our search criteria identified 4257 citations, of which 2568 were unique records (Figure 1). We excluded 1393 records in our initial screening at the title and abstract level. The majority of records excluded at this stage were articles that did not present empirical findings (e.g. commentary, editorial letters, study protocols), did not include transgender participants, did not focus on topics related to HIV or HIV prevention, or centred on treatment as prevention. We also excluded review papers and seven articles for which the full text was not available. Following an assessment of the full text of the remaining 1175 articles, we excluded 508 records that did not meet the inclusion criteria and included 667 articles (see Additional file 1 for a list of all included articles).

### 3.1 Transgender people as participants in HIV prevention research: Types of inclusion

Regarding our classification of the ways that transgender people were included as participants in HIV prevention research (Table 1), we found that 39% of studies subsumed transgender participants into other populations (*n =* 256). Studies in this category had the lowest number of transgender participants – ranging from 1 to 4420, with over half having fewer than 25 transgender participants and only 17% having 100 transgender participants or more. Similarly, these studies had the lowest percentage of transgender participants over the total sample size, ranging from less than 1%–77%, with transgender participants representing less than 10% of all participants in more than half of these studies. The majority of studies in this category used sex assigned at birth as criteria to combine transgender women with cisgender MSM (*n =* 206; 80%).

**Table 1:** Sample size and percentage of transgender participants in total and by type of participation

		Type of participation^a^		
	Total 667 (100%)	Trans‐subsumed 256 (38.5%)	Trans‐comparative 136 (20.5%)	Trans‐exclusive 273 (41.1%)
**Total sample size**				
Median	250	345	554	137
IQR	75–595	168–700	116–2306	46–293
Range	8–9,303,616	10–38,586	12–9,303,616	8–3878
**Trans sample size**				
Median	59	23	101	137
IQR	19–200	7–67	28–285	46–293
Range	1–15,518	1–4420	4–15,518	8–3878
**% Trans/total sample**				
Median	31.3%	9.0%	20.0%	100%
IQR	9.6%–100%	3.3%–17.8%	10.7%–32.4%	n/a
Range	0.2%–100%	0.2%–76.7%	0.2%–68.0%	n/a

***Note***: ^a^Totals for columns by type of transgender participation do not add to 667 because two studies (using couple‐based research) were not classified into either type.

Abbreviation: IQR, interquartile range.

Studies grouping transgender participants and comparing them with cisgender groups were the least common (*n =* 136; 20%). Studies in this category varied greatly by number of transgender participants, ranging from 4 to 15,518. Half of these studies included less than 100 transgender participants (*n =* 67). The percentage of transgender participants over the total sample size ranged from less than 1%–68%. Unlike studies that subsumed transgender participants into other populations, transgender participants represented more than 10% of all participants in 76% of comparative studies (*n =* 104). Of the studies in this category, 77 (57%) focused on comparing transgender women with cisgender MSM. Comparisons of transgender women sex workers with cisgender (both men and women) sex workers were represented in 23 (17%) of articles.

The remaining articles included in this review sampled transgender participants exclusively (*n =* 273; 41%). Sample size for these studies ranged from 8 to 3878 participants, with 59% including 100 participants or more. Two couples‐focused studies, in which participants were transgender women and their cisgender main partners, were not classified in any of the three categories of transgender inclusion [39, 40]. In the following sections, these two studies are included in global descriptions, but are excluded from analysis focusing on type of transgender participation.

### 3.2 Transgender people as participants in HIV prevention research: Analyses by time, place, thematic areas, methods and gender identity of participants

#### 3.2.1 Chronological analysis

**Figure 2:**
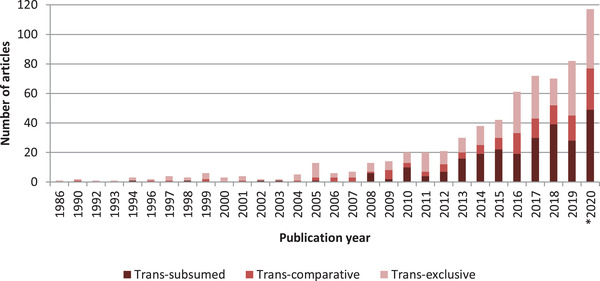
Publication year HIV prevention studies including transgender participants. *Note*: *Including eight articles published in 2021 that were available online ahead of print.

Figure 2 shows that the number of HIV prevention studies including transgender participants has increased over time, with over half published between 2017 and 2020 (51%). The oldest study included was a 1986 paper assessing AIDS awareness amongst transsexual sex workers in Singapore [41]. Twenty articles were published in the 1990's decade. By comparison, 117 were published in 2020 alone. When analysed by type of participation of transgender people (Table 2), studies published before 2011 were more likely to be trans‐exclusive (1986–2000: 73%; 2001–2010: 52%), whereas studies from 2011 to 2020 were more likely to subsume transgender participants into other populations (42%; χ^2^ = 23.71, df = 4; *p <*.001).

**Table 2:** Type of transgender participation in HIV prevention research: Variations over time, place, thematic areas, methods and gender identities of participants

		Type of participation		
	Total 667^a^ (100%)	Trans‐subsumed	Trans‐comparative	Trans‐exclusive
	256 (38·5%)	136 (20.5%)	273 (41·1%)
**Historic analysis (**χ^2^ **= 23.71, df = 4, *p* < .001)**				
1986–2000	26 (3.9%)	2 (7.7%)	5 (19.2%)	19 (73.1%)
2001–2010	86 (12.9%)	21 (24.4%)	20 (23.3%)	45 (52.3%)
2011–2020	555 (83.2%)	233 (42.1%)	111 (20.1%)	209 (37.8%)
**Geographic analysis (**χ^2^ **= 66.20, df = 12, *p* < .001)**				
Eastern and Southern Africa	25 (3.7%)	17 (68.0%)	4 (16.0%)	4 (16.0%)
Western and Central Africa	14 (2.1%)	10 (71.4%)	3 (21.4%)	1 (7.1%)
Asia and Pacific	150 (22.5%)	49 (32.7%)	42 (28.0%)	59 (39.3%)
Eastern Europe and Central Asia	0 (0%)	0 (0%)	0 (0%)	0 (0%)
Latin America and the Caribbean	101 (15.1%)	35 (34.7%)	27 (26.7%)	39 (38.6%)
Middle East and North Africa	6 (0·9%)	0 (0%)	0 (0%)	6 (100%)
USA	293 (43.9%)	17 (39.5%)	10 (23.3%)	16 (37.2%)
Western and Central Europe and Canada	43 (6.4%)	104 (35.7%)	41 (14.1%)	146 (50.2%)
Multiregional research	35 (5·2%)	24 (68.6%)	9 (25.7%)	2 (5.7%)
**Thematic analysis (**χ^2^ **= 62.86, df = 12, *p* < .001)**				
Psychological/Behavioural	70 (10.5%)	20 (29.0%)	10 (14.5%)	39 (56.5%)
Biomedical/Epidemiological	21 (3.1%)	12 (57.1%)	7 (33.3%)	2 (9.5%)
Social‐Structural	21 (3.1%)	6 (28.6%)	7 (33.3%)	8 (38.1%)
Psychological/Behavioural and Biomedical/Epidemiological	144 (21.6%)	82 (56.9%)	27 (18.8%)	35 (24.3%)
Psychological/Behavioural and Social‐Structural	167 (25·0%)	45 (27.1%)	26 (15.7%)	95 (57.2%)
Biomedical/Epidemiological and Social‐Structural	19 (2.8%)	10 (52.6%)	4 (21.1%)	5 (26.3%)
All three areas	225 (33.7%)	81 (36.0%)	55 (24.4%)	89 (39.6%)
**Methodological analysis (**χ^2^ **= 13.49, df = 4, *p* = .009)**				
Quantitative research	491 (73.6%)	33 (28.2%)	23 (19.7%)	61 (52.1%)
Qualitative research	117 (17·5%)	199 (40.7%)	107 (21.9%)	183 (37.4%)
Mixed‐methods research	59 (8.8%)	24 (40.7%)	6 (10.2%)	29 (49.2%)
**Gender identity analysis**^b^ (χ^2^ **= 45.04, df = 6, *p* < .001)**				
Transfeminine	496 (74.4%)	177 (35.8%)	111 (22.5%)	206 (41.7%)
Transmasculine	24 (3·6%)	5 (20.8%)	3 (12.5%)	16 (66.7%)
Gender diverse	105 (15.7%)	41 (39%)	14 (13.3%)	50 (47.6%)
Not specified	42 (6.3%)	33 (78.6%)	8 (19%)	1 (2.4%)

***Note***: ^a^Totals for columns by type of transgender participation do not add to 667 because 2 studies (using couple‐based research) were not classified into either type. ^b^Transfeminine refers to people assigned male sex at birth who identify on the feminine continuum (e.g. as women, trans‐women, male‐to‐female transgender); transmasculine describes those assigned female sex at birth who identify on the masculine continuum (e.g. as men, trans‐men, female‐to‐male transgender); gender diverse studies included transfeminine and/or transmasculine and/or gender expansive participants (e.g. gender non‐binary, a gender).

Abbreviation: df = Degrees of freedom.

#### 3.2.2 Geographic analysis

**Figure 3:**
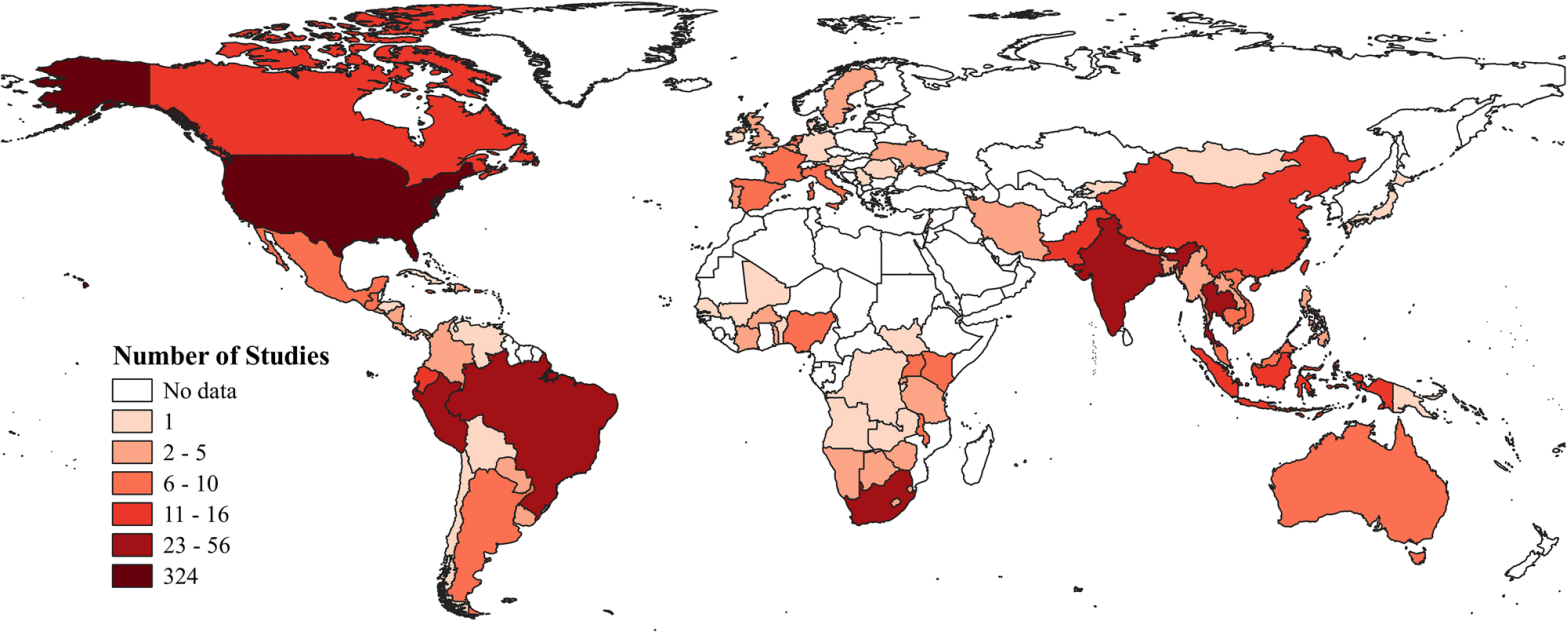
Countries in which HIV prevention research including transgender participants has been conducted. *Note*: One study with global data (respondents in 154 countries) is not included the map, because the article did not include the list of all countries from which participants came.

Figure 3 presents the countries where HIV prevention research that includes transgender participants has been conducted. Research was most prevalent in the USA (*n =* 324; 49%), followed by Thailand (*n =* 56; 8%), Peru (*n =* 53; 8%), Brazil (*n =* 44; 7%) and South Africa (*n =* 30; 4%). Forty‐five articles presented research conducted in more than one country, with almost a third of them resulting from the iPrEx study [38] (*n =* 14), and one based on the Global Men's Health and Rights online survey, with participants from 154 countries [42]. Regarding UNAIDS regions, 35 articles presented data from studies conducted in more than one region (5%). Amongst studies conducted in a single region, we did not find any study from ‘Eastern Europe and Central Asia’, and found six studies conducted in the ‘Middle East and North Africa’ (MENA) region. There were significant regional differences in the way transgender participants have been included in HIV prevention research (χ^2^ = 66.2; df = 14; *p* < .001). As presented in Table 2, multiregional studies and those conducted in Africa (except North Africa) were the most likely to subsume transgender participants into other populations and the least likely to include exclusively transgender samples. The six studies conducted in the MENA region included transgender samples only. Studies conducted in the USA were the least likely to be trans‐comparative (*n =* 41; 14%), whereas about a fourth of studies conducted in the ‘Asian and Pacific’ (*n =* 42; 28%) and ‘Latin America and the Caribbean’ (*n =* 27; 27%) regions fell in this category.

#### 3.2.3 Thematic analysis

**Figure 4:**
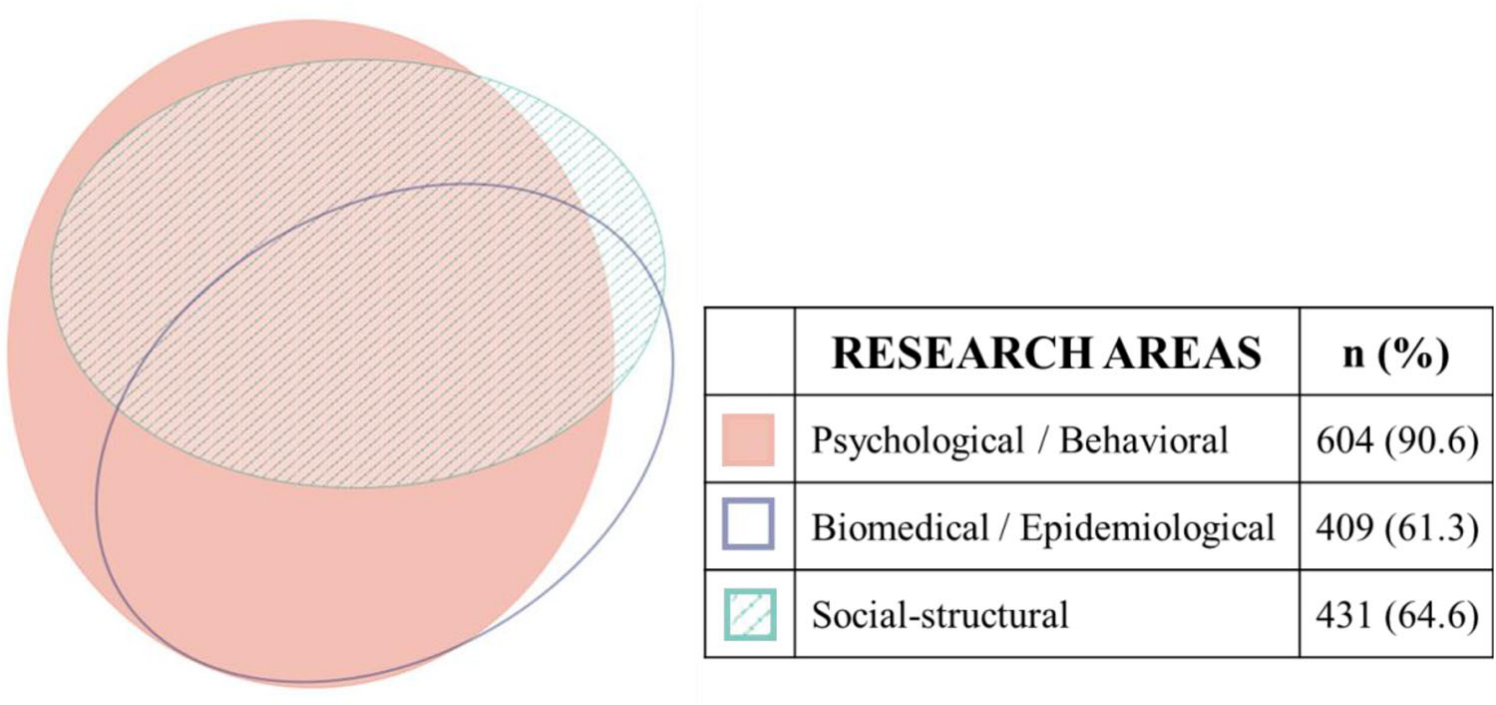
Venn diagram – areas in HIV prevention research including transgender participants. (Created using eulerAPE, http://www.eulerdiagrams.org/eulerAPE [43]).

Most of the studies reviewed covered psychological/behavioural topics (*n =* 604; 91%), and over half presented findings on biomedical/epidemiological (*n =* 409; 61%) or social‐structural (*n =* 431; 65%) research. Figure 4 shows that there was considerable overlap amongst research areas, with 80% of articles covering more than one area (*n =* 380). The most common combination was articles including all three areas (*n* = 225; 34%), followed by articles including both psychological/behavioural and social‐structural research (*n =* 166; 25%); 19 articles included biomedical/epidemiological and social‐structural topics (3%). Of the articles covering a single area, 69 focused on psychological/behavioural (10%), 21 on social‐structural (3%) and 21 on biomedical/epidemiological research (3%).

There were significant differences across research areas in the ways in which transgender participants were included (χ^2^ = 62.9; df = 12; *p* < .001). Of studies covering psychological/behavioural and social‐structural areas, and those focusing on psychological/behavioural research only, over half included transgender participants only, whereas less than a quarter of biomedical/epidemiological studies (alone or in combination with psychological/behavioural research) were trans‐exclusive. Trans‐comparative studies were most common in research focused on social‐structural or biomedical/epidemiological issues (33%). Subsuming transgender participants into other groups was particularly common in studies focusing on biomedical/epidemiological topics alone (57%) or in combination with one other area (psychological/behavioural: 57%; social‐structural: 53%).

#### 3.2.4 Methodological analysis

Most studies included in this review used quantitative (*n =* 491; 74%), followed by qualitative (*n =* 117; 17%) and mixed methods research (*n =* 59; 9%). Quantitative studies were least likely to be trans‐exclusive (*n* = 183; 37%), qualitative studies were least likely to be trans‐subsumed (*n* = 33; 28%) and mixed‐methods research least likely to be trans‐comparative (*n* = 6; 10%; χ^2^ = 13.49; df = 4; *p* = .009).

#### 3.2.5 Analysis by gender identity

The vast majority of studies included transgender women only (*n =* 496; 74%), whereas fewer included transgender men only (*n =* 24; 4%). Over 100 studies included transgender women and/or men as well as gender expansive participants (*n =* 105; 16%). The remaining 42 studies did not specify the gender identities of participants (6%). Studies with transmasculine participants only were more likely to be trans‐exclusive (*n* = 16; 67%). Studies that did not specify the gender identities of participants were more likely to be trans‐subsumed (*n =* 33; 79%; χ^2^ = 45.04; df = 6; *p* < .001).

### 3.3 Summary of findings

Results from this scoping review show that inclusion of transgender people in HIV prevention research has increased globally, especially since 2012, after several calls to recognize transgender people as a unique population in HIV research [7, 8, 9, 10, 11]. Trans‐centred research represented the majority of the papers in this review, with 273 trans‐exclusive and 136 trans‐comparative studies. Trans‐subsumed research, however, has not disappeared in HIV prevention research, and was actually the most common approach in the most recent decade (2011–2020). Our scoping review also shows that the ways in which transgender people are included in HIV prevention research vary greatly over time, place, thematic areas and methodological approaches. Transgender women are the focus of the majority of reviewed studies, whereas transgender men and gender expansive people are rarely included as participants in this research.

Table 3 presents a summary of the advantages and challenges associated with the three types of transgender participation, and recommendations for when to use each of them. It is also possible to combine types of participation. For example, researchers who have trans‐subsumed data sets can contribute to trans‐centred research by conducting secondary analysis that either focus exclusively on transgender participants or compare them with other groups [44, 45]. If sample size is too small for meaningful analyses, researchers may consider reporting trans‐centred findings as supplemental material, so they can be included in future meta‐analyses and used as preliminary data for trans‐centred grant applications. Researchers who have comparable variables may also pool data sets to facilitate comparisons over time and place.

**Table 3:** Advantages, challenges and recommendations for use of each type of transgender participation in HIV prevention research

	Type of participation		
	Trans‐subsumed	Trans‐comparative	Trans‐exclusive
Advantages	Does not require large numbers of transgender participants.	Allows detecting health inequities and understanding the factors associated with those inequities.	Allows a deeper understanding of the factors associated with HIV prevention amongst transgender populations. This approach is particularly relevant given the epidemiological evidence of disproportionate HIV burden amongst transgender women and transgender men who have sex with cisgender men.
Challenges	Assumes that results are equally applicable to transgender and cisgender participants based on a common element, such as their sex assigned at birth.	Requires large numbers of transgender participants to allow multi‐group comparisons, particularly for quantitative papers.	Risk ‘othering’ transgender people, particularly if cisnormative standards (i.e. the notion that all people are cisgender) remain unquestioned [52].
Recommended for	When there are no differences in the prevalence of the HIV prevention outcomes or in the biological, behavioural, psychological, social and structural factors associated with them.	Identifying elements to consider when developing interventions for transgender populations based on those originally developed for cisgender groups [46].	Identifying transgender‐specific HIV prevention needs and developing or adapting interventions that respond more effectively to those needs [46].

### 3.4 Moving from trans‐subsumed to trans‐centred research

Transgender people have participated in HIV prevention research since the beginning of the HIV epidemic, but many earlier studies failed to identify them as a distinct group. Researchers are becoming increasingly aware of the relevance of – at the very least – including, identifying and enumerating transgender participants in their studies. Despite their greater inclusion, considerable gaps in knowledge remain about HIV prevention amongst transgender people. Although trans‐subsumed studies are an improvement over research that excludes or ignores transgender populations, an underlying assumption of trans‐subsumed research is that results are equally applicable to transgender and cisgender participants based on a common element, such as their sex assigned at birth, the sex of their sexual partners or their engagement in transactional sex. For instance, 80% of trans‐subsumed studies used sex assigned at birth to subsume transgender women into the MSM category. This practice undermines transgender women's womanhood and the research findings may not provide appropriate recommendations for them [46].

The case of oral PrEP provides a good example of the potential limitations of the underlying assumption of trans‐subsumed research: Compared to the 44% effectiveness in the iPrEx trial [38], a sub‐group analysis of transgender women participating in the trial (14% of the total sample) indicated no effectiveness on an intention‐to‐treat basis for this group [44]. The lack of effectiveness was associated with reduced tenofovir concentrations in blood amongst transgender women compared with cisgender MSM [44]. Three recent trans‐centred studies reported that the use of feminizing hormones was associated with reduced PrEP concentrations in blood, suggesting that the reduced efficacy of oral PrEP is not merely the result of lower adherence amongst transgender women [47, 48, 49]. Another recent study assessing the drug interactions between sex hormone therapy and oral PrEP showed that tenofovir concentrations were comparable between transgender women and cisgender men, but not between transgender men and cisgender women, although all participants were projected to reach protective PrEP levels [50]. Furthermore, a secondary analysis of iPrEx trial data showed that significant differences in baseline characteristics between transgender women and cisgender MSM (e.g. depression, transactional sex in the last 6 months) explained the heterogeneity of oral PrEP efficacy [51]. These findings show that we need to go beyond simply including transgender participants in research to increase our understanding of the *specific* needs and characteristics of transgender people. Failing to attain a deeper understanding has serious implications for HIV prevention in a group at elevated risk for HIV.

Trans‐centred studies intentionally ask specific questions regarding transgender people, either by focusing exclusively on them (i.e. trans‐exclusive) or by comparing them with cisgender groups (i.e. trans‐comparative). Our scoping review found that trans‐exclusive studies were the most common and trans‐comparative the least common. A potential limitation of trans‐centred research is the risk of ‘othering’ transgender people, particularly if cisnormative standards (i.e. the notion that all people are cisgender) remain unquestioned [52]. To avoid this ‘othering’, it is important to frame trans‐centred studies within critical frameworks – such as intersectionality or critical gender and critical race theories – which expose the social structures and processes that generate and maintain inequality for transgender populations at diverse intersections of race, class, gender expression and sexual minority status [53]. For example, when reporting a high prevalence of transactional sex, researchers should explicitly acknowledge that the legal structures (e.g. criminalization of transgender status, lack of non‐discriminatory employment laws) and the exclusionary and transphobic workplaces are drivers towards sex work for many transgender people, whereas sex work is an occupational choice for others.

### 3.5 Recommendations for future HIV prevention research

Echoing previous reviews and conceptual papers on transgender health [3, 4, 5, 12, 13, 14, 15, 16, 17, 18, 19, 20, 21, 22, 23, 24, 25, 26, 27, 28, 46, 54, 55], we suggest the following recommendations to move from trans‐subsumed to trans‐centred research:

1. *Include transgender people as participants in research*. It is frequently argued that transgender populations are too small for inclusion in HIV prevention trans‐centred research. Almost 30 million people currently identify as transgender, and this number will continue to increase as transgender people are able to come out more safely [56]. Furthermore, the size of a population is not an indicator of its importance and is a poor criterion for research inclusion. Transgender populations are unique in multiple ways and face a disproportionate burden of HIV that warrants their inclusion in HIV prevention research [57].
2. *Identify transgender participants using multi‐step methods*. There are two typical ways of collecting information about gender identity in HIV prevention research. The first one is to ask a single question about gender identity (e.g. woman, man or transgender). The second one is a multi‐step method that asks separately about sex assigned at birth and current gender identity [58, 59]. A study that compared these two approaches found that the single‐item measure missed 70% of transfeminine and 40% of transmasculine participants identified with a multi‐step method [60]. Thus, asking about sex assigned at birth and current gender identity separately identifies transgender participants more accurately. When using multi‐step methods in new settings, we recommend cultural adaptation of the items. Moreover, to capture gender fluidity and changes in gender identity over time, we recommend including two‐step questions at all data collection times in longitudinal studies.
3. *Acknowledge that transgender people are not a monolithic group*. Consider factors associated with differential outcomes, such as gender identity, gender expression, sexual orientation, race/ethnicity and stage in gender affirmation process. In terms of gender identity, for example, over 75% of the studies in this review included transfeminine participants only. Although HIV disproportionally affects transgender women, the few studies that include transgender men show that this population, particularly those who have sex with cisgender men, are also at higher risk for HIV than the general population [4]. Unfortunately, data on HIV risk amongst gender expansive people are even more limited [61]. In addition to the individual‐level variability amongst transgender people, it is also important to recognize that the social‐structural context in which transgender people live varies at multiple levels (e.g. country, city, neighbourhood) and has a profound impact on their lives and health.
4. *Make research trans‐affirming and actively engage transgender people at all stages*. Trans‐friendly research starts by listening to the community and involving transgender people as part of the research teams and/or as members of community advisory boards [54, 62]. Community‐based or other participatory research approaches are particularly apt for conducting trans‐centred research and provide a forum to disseminate research findings back to the communities. There is an urgent need to nurture the pipeline of transgender researchers, so that HIV prevention research that includes transgender people can be trans‐grounded and trans‐driven [63]. Because this pipeline is still developing, it is also important to strengthen the training of cisgender researchers and research staff on transgender health issues and transgender cultural competence. The Cross‐Network Transgender Working Group from the USA National Institutes of Health developed a series that is an excellent training source [64]. Lastly, multidisciplinary teams are needed to identify and intervene on the multilevel factors associated with heightened HIV prevalence amongst transgender populations, including psychological/behavioural (e.g. need for gender affirmation, sexual positioning), social‐structural (e.g. stigma, lack of non‐discrimination laws) and biomedical (e.g. role of hormone use, sex reassignment surgeries).

Researchers who already have trans‐subsumed data sets can also contribute to trans‐centred research, by conducting secondary analysis that either focus exclusively on transgender participants or compare them with other groups [44, 45]. If sample size is too small for meaningful analyses, researchers may consider reporting trans‐centred findings as supplemental material, so that they can be included in future meta‐analyses and used as preliminary data for trans‐centred grant applications. Researchers who have comparable variables may also pool data sets to facilitate comparisons over time and place.

Our review is not without limitations. Although our literature search was not limited to articles in English, our key words were in English. Therefore, we may have excluded articles in other languages. This limitation is particularly important when interpreting the geographical analysis. We excluded grey literature (e.g. conference abstracts), which may provide a more comprehensive overview of available evidence. Lastly, our thematic classification was very broad, particularly in the social‐structural area. Most of the studies classified as social‐structural merely included socio‐demographic information (e.g. income, health insurance) as control variables. Few studies delved on the role of social‐structural issues such as stigma and discrimination, incarceration, violence and community mobilization.

## 4 CONCLUSIONS

The purpose of this scoping review was to characterize the inclusion of transgender people as participants in HIV prevention research. Several systematic reviews, meta‐analyses and other literature overviews have reviewed HIV‐related research with transgender participants [3, 4, 5, 12, 13, 14, 15, 16, 17, 18, 19, 20, 21, 22, 23, 24, 25, 26, 27, 28]. This scoping review adds to this literature by focusing on the ways in which transgender people are included as participants in HIV prevention research. Our scoping review shows that inclusion is increasing, but several gaps remain, particularly in the inclusion of transgender men and gender expansive people as participants, and the limited focus on social‐structural factors that increase HIV vulnerability amongst transgender populations. We must use this momentum to move from trans‐subsumed to trans‐centred research that enables us to better understand and respond to HIV prevention needs, reduce HIV infections and improve health outcomes amongst transgender people.

## COMPETING INTERESTS

The authors declare that they have no competing interests.

## AUTHORS’ CONTRIBUTIONS

AMdRG developed the initial concept for the manuscript, designed the Review, did the literature search, led inclusion decisions and data abstraction, created figures and tables, interpreted the literature and led writing of the manuscript. MLF, DM, RAR and CG participated in inclusion decisions, data abstraction and revisions of the manuscript. LB and MCZ contributed to the writing and revision of the manuscript. All authors reviewed and approved the final version of the manuscript for submission.

## ADDITIONAL FILES

Additional file 1:

## LIST OF ARTICLES INCLUDED IN THE REVIEW

Format: Excel spreadsheet. Contents: References of all articles included in the review, and classification regarding type of inclusion of transgender participants (i.e. trans‐subsumed; trans‐comparative; trans‐exclusive).

## Supporting information

SUPPORTING INFORMATION
